# Utilising the native plasmid, pCA2.4, from the cyanobacterium *Synechocystis* sp. strain PCC6803 as a cloning site for enhanced product production

**DOI:** 10.1186/s13068-015-0385-x

**Published:** 2015-12-01

**Authors:** Patricia Armshaw, Dawn Carey, Con Sheahan, J. Tony Pembroke

**Affiliations:** Molecular and Structural Biochemistry Laboratory, Department of Chemical and Environmental Sciences; Materials and Surface Science Institute, University of Limerick, Limerick, Ireland; Department of Manufacturing and Operations Engineering, Faculty of Science and Engineering, University of Limerick, Limerick, Ireland

**Keywords:** Yellow fluorescent protein (YFP), pCA2.4, Gene dosage, Gene expression, *Synechocystis* PCC6803

## Abstract

**Background:**

The use of photosynthetic autotrophs and in particular the model organism *Synechocystis* PCC6803 is receiving much attention for the production of sustainable biofuels and other economically useful products through metabolic engineering. Optimisation of metabolic-engineered organisms for high-level sustained production of product is a key element in the manipulation of this organism. A limitation to the utilisation of metabolically-engineered *Synechocystis* PCC6803 is the availability of strong controllable promoters and stable gene dosage methods for maximising gene expression and subsequent product formation following genetic manipulation.

**Results:**

A native *Synechocystis* PCC6803 small plasmid, pCA2.4, is consistently maintained at a copy level of up to 7 times that of the polyploid chromosome. As this plasmid is stable during cell division, it is potentially an ideal candidate for maximising gene dosage levels within the organism. Here, we describe the construction of a novel expression vector generated from the native plasmid, pCA2.4. To investigate the feasibility of this new expression system, a yellow fluorescent protein (YFP) encoding gene was cloned downstream of the strong Ptrc promoter and integrated into a predicted neutral site within the pCA2.4 plasmid. The stability of the integrated construct was monitored over time compared to a control strain containing an identical YFP-expressing construct integrated at a known neutral site in a chromosomal location.

**Conclusions:**

A significantly higher fluorescence level of the yellow fluorescent protein was observed when its encoded gene was integrated into the pCA2.4 native plasmid when compared to the isogenic chromosomally integrated control strain. On average, a minimum of 20-fold higher fluorescence level could be achieved from integration into the native plasmid. Fluorescence was also monitored as a function of culture time and demonstrated to be stable over multiple sub-cultures even after the removal of selective pressure. Therefore, the native small plasmid, pCA2.4 may be utilised to stably increase gene expression levels in *Synechocystis* PCC6803. With the complementary utilisation of an inducible promoter system, rapid generation of commodity-producing *Synechocystis* PCC6803 strains having high level, controlled expression may be more achievable.

**Electronic supplementary material:**

The online version of this article (doi:10.1186/s13068-015-0385-x) contains supplementary material, which is available to authorized users.

## Background

Reserves of fossil fuels are declining while greenhouse gas emissions are on the rise [1]. This has led to an increasing emphasis on the research of biofuel production systems that harness CO_2_. For this reason, interest in the utilisation of cyanobacteria for biotechnological applications has been increasing [2]. Cyanobacteria have the capacity to convert carbon dioxide to organic carbon metabolites and through genetic modification of these organisms; these metabolites can be converted into commodities for biofuel applications [3, 4].

Currently, industrial utilisation of such modified organisms to produce biofuels and other commodities are uneconomic due to the high cost of production and low titers often initially produced [3]. Consequently, efforts to utilise cyanobacteria economically require significant increases in production rates of the target product [4]. *Synechocystis* sp. strain PCC6803, henceforth referred to as *Synechocystis* PCC6803, is commonly used as a model cyanobacterium due to its relative ease of genetic manipulation; its ability to grow both autotrophically and heterotrophically with minimal light; and ability to undergo natural transformation [5]. In addition, this strain can express several well-characterised *Escherichia coli* promoters, although with altered control in many cases [6]. Tightly regulated *E. coli* promoters can be leakier and of lower strength in *Synechocystis* PCC6803 [7]. In addition, *Synechocystis* PCC6803 is polyploid and the slow growth rate of the strain can significantly lengthen the time required for any genetic modification [8]. In spite of these recognised difficulties, *Synechocystis* PCC6803 has been shown to have the potential to produce many economically interesting products. For a detailed review, please see Dexter et al. 2015 [3] and Angermayr et al. 2015 [4]. However, the issue of initial low production levels of target compounds of interest is a limiting concern [9].

A general method for increasing the production of target compounds is to alter the expression levels of the specific genes required for product formation to increase the carbon flux directed towards the commodity production [10]. Indeed, if the expression level of the necessary enzymes is not in excess, other methods such as increasing the carbon flux coming into the cell will likely not lead to increased commodity titers and the increased carbon flux is likely to be directed towards biomass formation [11]. Studies have found that utilising multiple copies of the necessary genes for production within the chromosome or known strong promoters can increase product titers [10, 12]. However, the generation of modified strains with multiple chromosomal insertions can have several drawbacks. With the slow growth rate of *Synechocystis* PCC6803, each separate modification can be time consuming to generate with multiple modifications requiring many rounds of cloning, transformation, segregation and selection especially if marker-less clones are required. Several different antibiotic cassettes can be required to build the constructs which can be of concern in pilot scale photobioreactors due to the cost and controversy concerning their use [13]. In addition, multiple different neutral sites are needed to provide multiple insertions. All this can be significantly time-consuming and multiple-cassette containing strains may be unstable and difficult to maintain, losing copies of the inserted cassettes over time [14]. Ideally, integration of a single cassette into a high-copy number plasmid that would be stably maintained within the cell may allow maximisation of gene expression while minimising the time required for strain generation. Utilisation of a native high-copy number plasmid that is naturally and consistently maintained could potentially be more stable than a high-copy number non-native plasmid that has been transformed into the strain.

pCA2.4 is a small cryptic native plasmid found to be consistently present in *Synechocystis* PCC6803 [15]. It is stably maintained within the cell indicating that the plasmid may provide some unknown essential function. It is 2378 bp in size and contains two predicted ORFs with little known about its function. One of the two ORFs, *repA*, has an annotated function that suggests the plasmid replicates by a rolling circle mechanism [15]. The copy number of this plasmid when analysed [16] was found to change significantly depending on growth phase and nutrient conditions utilised. Under autotrophic conditions, the plasmid copy number per chromosome during exponential phase was approximately 0.75 changing to 5.41 during stationary phase. Utilisation of mixotrophic conditions (5 mM glucose) led to higher copy numbers during both phases of growth: 6.26 (exponential) and 7.39 (stationary) [16].

As pCA2.4 is stably maintained within *Synechocystis* PCC6803 without any recognisable selective pressure, integration of a production cassette within the plasmid, if stable, would have the potential to increase the gene copy number up to 7 times higher than that of a chromosomal integration site. In addition, the expression level of the plasmid could be controlled by modification of the growth conditions or by utilisation of promoters of varying strength. Modified strains could be more easily generated by carrying out transformation and strain segregation under low plasmid copy number conditions. Maximising expression could be achieved by maintaining the cell culture at stationary phase, under mixotrophic conditions if desired or by the utilisation of an inducible promoter system such as a riboswitch [17]. Several Ptrc riboswitches have recently been characterised in cyanobacteria and tightly regulated expression shown to be possible [18]. However, the maximal expression levels of the Ptrc promoter with a riboswitch are reduced indicating that gene dosage with a riboswitch system would be necessary for reasonable production rates to be achieved [18].

To investigate the possibility of utilising this native plasmid as a stable neutral site for maximising gene expression within *Synechocystis* PCC6803, several constructs, including one that contained a yellow florescent protein encoding gene under the control of a constitutive Trc promoter (Ptrc), were generated (Table 1). These constructs were integrated at both a chromosomal (*psbA2*) and a putative pCA2.4 neutral site location. The relative fluorescence levels were extensively examined to determine if pCA2.4 could be utilised to express high levels of desired genes under long-term culturing conditions.

**Table 1:** Bacterial strains, genetic elements, plasmids and primers used in this study

Strain	Genotype/phenotype	Source
TOP10	*E. coli* strain. F^−^, *mcrA0*, ∆(*mrr*-*hsdRMS*-*mcrBC*), φ80d*lacZ58*(M15), ∆*lacX74*, *recA1*, *araD139*, ∆(*araA*-*leu*)*7697*, *galU* ^−^, *galK0*, *rpsL* ^−^ (Str^R^), *endA1*, *nupG* ^−^	Bio-Sciences, Dun Laoghaire, Dublin, Ireland
AA314	Wild-type *Synechocystis* PCC6803 strain	K. Hellingwerf, UvA, Amsterdam, The Netherlands
UL006	PCC6803 transconjugant with Ptrc-YFP, *psbA2* locus, Kan^R^	This study
UL007	PCC6803 transconjugant with Ptrc-YFP-E*, *psbA2* locus, Kan^R^	This study
UL008	PCC6803 transconjugant with P*psbA2*-YFP, *psbA2* locus, Kan^R^	This study
UL018	PCC6803 transconjugant with pCA2.4-Ptrc-YFP, Kan^R^	This study
UL022	PCC6803 transconjugant with Ptrc-GAL, *psbA2* locus, Kan^R^	This study
UL023	PCC6803 transconjugant with Ptrc-GAL-E*, *psbA2* locus, Kan^R^	This study

Plasmid/ICE	Genotype/phenotype	Source
pEYFP	YFP source plasmid	Clontech, CA 94043, USA
pSF-OXB20-BetaGal	β-galactosidase gene source plasmid	Oxford Genetics Ltd, Oxfordshire, OX25 5HD, UK
ICE R391	Kanamycin resistance gene source Integrative conjugative element	[26]
pUC18	Am^R^ backbone plasmid	Sigma-Aldrich, Arklow, Wicklow, Ireland
pCR2.1-pTRC-riboswitchE*	pTRC-E* and pTRC source plasmid	Eurofins mwg/operon, 85560 Ebersberg, Germany
P*psbA2*-YFP	P*psbA2* promoter, YFP, *psbA2* integration site, Kan^R^	This study
Ptrc-YFP	Ptrc promoter, YFP, *psbA2* integration site, Kan^R^	This study
Ptrc-YFP-E*	Ptrc promoter, YFP, riboswitch E*, *psbA2* integration site, Kan^R^	This study
pCA2.4-Ptrc-YFP	Ptrc promoter, YFP, pCA2.4 integration site, Kan^R^	This study
P*psbA2*-GAL	P*psbA2* promoter, β-galactosidase, *psbA2* integration site, Kan^R^	This study
Ptrc-GAL	Ptrc promoter, β-galactosidase, *psbA2* integration site, Kan^R^	This study
Ptrc-GAL-E*	Ptrc promoter, β-galactosidase, riboswitch E*, *psbA2* integration site, Kan^R^	This study

## Results and discussion

### Selection of a suitable reporter gene and promoter construct

**Fig. 1:**
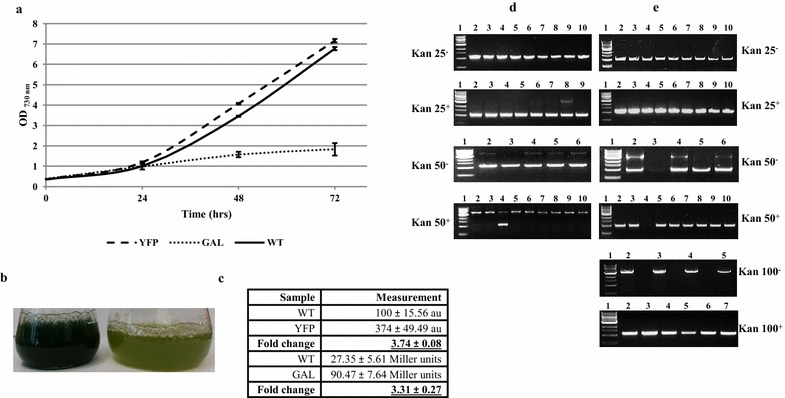
Determination of an optimal reporter gene in *Synechocystis* PCC6803; optimisation of integration into plasmid pCA2.4. **a** Growth rate comparison for *Synechocystis* PCC6803 strains expressing yellow fluorescent protein (YFP, UL006) and β-galactosidase (GAL, UL022) under the control of the constitutive Ptrc promoter in a chromosomal integration site (psbaII). Note: the strain constitutively expressing β-galactosidase displays significant growth retardation. **b** UL006 (YFP) 72 h culture on the *left*; UL022 (GAL) 72-h culture on the *right*. Note: the poor *green colour* of the strain constitutively expressing β-galactosidase. **c** Expression of both reporter genes under the constitutive Ptrc promoter results in a similar fold increase in expression measurements compared to wild-type [3.74 fold (YFP, au) versus 3.31 fold (GAL, Miller units)]. **d** PCR screening of Ptrc-YFP integration within the chromosomal psbaII site. PCR primers utilised flanked either side of the targeted site of integration (psbaII site) of the transformed construct Ptrc-YFP. Lower ~1 kb band represents WT and higher ~4 kb represents integration of cassette. Increasing kanamycin concentration and inclusion of glucose allowed rapid generation of a segregated YFP-expressing strain. **e** Integration of the same cassette within pCA2.4 required higher concentration of kanamycin and sub-culture to liquid media and inclusion of glucose prevented detectable integration. Note: + with glucose, - no glucose. Again note the PCR primers utilised flanked either side of the targeted site of integration (predicted pCA2.4 neutral site, Additional file 1: Figure S1). Lower ~1 kb band represents WT and higher ~4 kb represents integration of cassette

The objective of this work was to determine if pCA2.4 could be utilised for stable high-level expression of genes of interest in *Synechocystis* PCC6803. To determine the true maximal gene expression level that can be reached with integration into pCA2.4 without toxicity effects, two reporter genes were considered (Fig. 1). While both yellow fluorescent protein [YFP] and β-galactosidase (GAL) expression were detectable at similar levels with a chromosomal integration (Fig. 1c), it was found that integration of the β-galactosidase gene within the chromosomal neutral site resulted in significant growth retardation (Fig. 1a). While β-galactosidase has previously been utilised in *Synechocystis* PCC6803 as a reporter gene [19, 20], in this case it was expressed under the control of a very strong constitutive promoter, Ptrc and as such the expression of β-galactosidase under the control of this promoter was found to be adverse. Integration of a β-galactosidase expressing construct into pCA2.4 could lead to even higher expression levels, hence it was determined that YFP, which displayed a similar growth rate to wild-type when expressed under the control of the Ptrc promoter within the chromosomal neutral site would be a more favourable reporter gene to be utilised throughout this analysis.

**Fig. 2:**
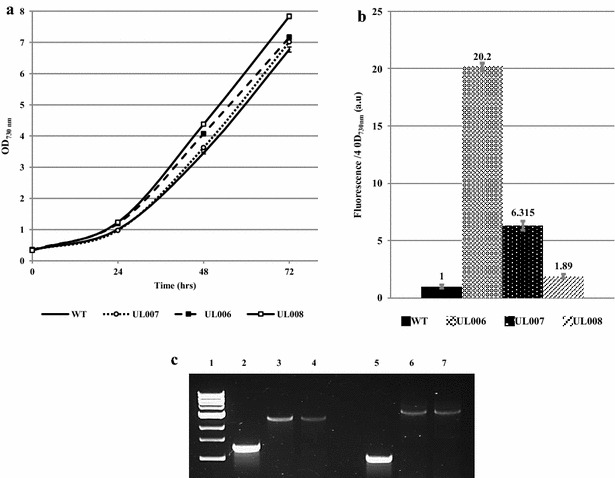
Relative fluorescence levels of screened promoters and stability of modified pCA2.4 strains. **a** Optical density measurements at OD_730nm_ of growth of different YFP-expressing strains over 72 h. Chromosomally integrated (psbA2 locus) YFP strains UL006 (Ptrc-YFP), UL007 (Ptrc-YFP-E*) and UL008 (PpsbA2-YFP) grow similarly to WT. **b** Measurement of UL006, UL007 and UL008 YFP fluorescence levels after 72 h. The highest YFP fluorescence level detected was in UL006. Note 2 mM of the inducer theophylline was utilised to induce expression of the Ptrc riboswitch in UL007. **c** UL018 and UL006 strain stability after 80 days culturing. *Lane 1* marker, *lane 2* wild-type, *lane 3* UL006 minus kanamycin, *lane 4* UL006 plus kanamycin, *lane 5* wild-type, *lane 6* UL018 minus kanamycin, *lane 7* UL018 plus kanamycin. The lower band indicates the presence of WT; higher band indicates construct integration. No evidence of the WT band in transconjugant strains was observed

Similarly, an initial screening with both β-galactosidase and YFP expressed under the control of different promoters was carried out with the aim of determining a promoter-gene construct that would give a strong detectable signal when integrated in the chromosomal control neutral site—*psbA2* (Fig. 2). Three promoter-gene combinations were trialled; Ptrc, Ptrc-E* and P*psba2*. Ptrc is a strong promoter that is IPTG inducible derived from the *Escherichia coli* trp and lacUV5 promoters [21]. In this case, the Ptrc promoter was modified to remove its repressor thereby allowing constitutive expression of the report gene. Ptrc-E* is a Ptrc promoter linked to a riboswitch (E*) that has been shown to allow inducible expression in *Synechococcus elongatus* PCC7942 through the use of the inducer compound theophylline [18]. P*psbA2* is a native *Synechocystis* PCC6803 light inducible promoter regularly utilised for expression of genes in *Synechocystis* PCC 6803 [22]. As can be seen (Fig. 2b), the highest level of fluorescence was observed with the Ptrc promoter. As noted earlier, the introduction of a riboswitch, while allowing inducible control of the Ptrc promoter in *Synechocystis* PCC 6803, even when induced, reduced the maximum fluorescence level of the promoter significantly from a potential 20.2 to 6.32 au (Fig. 2b). In addition, it was found that the regularly utilised P*psba2* promoter expressed YFP at a significantly lower level than the constitutive Ptrc promoter: 1.89 au compared to 20.2 au. Hence, a Ptrc-YFP combination was determined as the optimal construct for integration within the chromosomal locus *psba2* and the predicted pCA2.4 plasmid neutral site (Fig. 2b).

### Optimisation of the transformation and segregation conditions

Introduction of foreign DNA into *Synechocystis* PCC6803 can be carried out via natural transformation [23]. As each cell contains multiple chromosome copies and non-native transformed plasmids can be lost during cell division, integration of foreign DNA into the chromosome is the preferred method. However, as the normal doubling time of the strain can be up to 20 h and each cell contains multiple copies of the chromosome, obtaining a fully segregated strain with a copy of the introduced DNA in each chromosome can take several weeks. In addition, several sub-cultures of the transformed colonies with increasing concentration of a selective pressure, usually a linked antibiotic resistance gene, is necessary for complete segregation. However, if a glucose tolerant sub-strain of *Synechocystis* PCC6803 is utilised [24], the incorporation of glucose into the nutrient media can increase the growth rate of the strain and minimise the time between sub-cultures. In this analysis, the generation of a fully segregated strain containing the Ptrc-YFP cassette within the *psbA2* neutral site took 28 days under autotrophic conditions and 16 under mixotrophic conditions. In each case, transformed colonies were initially cultured on 5 µg ml^−1^ kanamycin and sub-cultured to 25 µg ml^−1^ and then 50 µg ml^−1^ before full segregation occurred. Without glucose, a second sub-culture on 50 µg ml^−1^ kanamycin was required to generate fully segregated strains.

Conversely, as the native plasmid pCA2.4 has an increased copy number under mixotrophic conditions [16], the incorporation of glucose prevented the generation of fully segregated strains. Figure 1e shows the screening of such transformant colonies. Note that in any conditions that incorporated glucose, no higher PCR band indicating the presence of Ptrc-YFP construct integration in the pCA2.4 plasmid predicted neutral site can be observed. In addition, likely due to the higher copy number of the plasmid relative to the chromosome, additional sub-cultures and a higher antibiotic concentration were necessary for complete segregation of pCA2.4 transformants. Several pCA2.4 transformation attempts were carried out to optimise the transformation process. It was found that multiple further sub-cultures (up to 4) at high antibiotic concentration (50–100 µg ml^−1^ kanamycin) in liquid media were necessary to achieve full segregation of pCA2.4 transformants (Fig. 1e).

As little is known about pCA2.4 and its function within the cell, the neutral site utilised within the plasmid may influence the ability to obtain a fully segregated stable strain [25]. Recently, Ng et al. [25] attempted to integrate a YFP cassette into 6 different predicted neutral sites within three different native plasmids of *Synechocystis* PCC6803. For 5 of these predicted neutral sites, detectable integration was not observed. For the 6th, within native plasmid pCC5.2, a semi-segregated strain was obtained. No integration within the native plasmid pCA2.4 was detected [25]. In our study, full segregation of modified pCA2.4 plasmids could be achieved. This could be due either to the additional sub-cultures being carried out at high antibiotic concentrations in liquid media or the utilisation of a different predicted neutral site to those attempted by Ng et al. [25]. Thus, the choice of the neutral site may be quite important. In the case of Ng et al. [25], the unsuccessful neutral sites chosen were either within a predicted promoter region (1747–1819 bp, GenBank ID: L13739.1) or close to a predicted promoter region (390–523 bp, GenBank ID: L13739.1) [15, 25]. This may have contributed to the inability to integrate into the plasmid and generate stable transformants.

### Expression levels achievable from pCA2.4 compared to chromosomal insertion

**Fig. 3:**
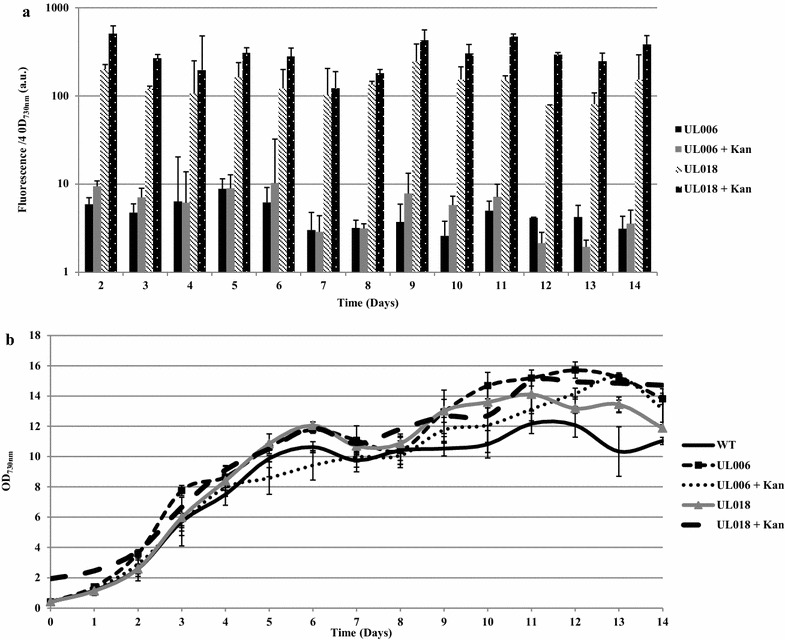
Stability of UL018 YFP fluorescence over 14 days without selective pressure. **a** Relative fluorescence measurements over 14 days for UL006 (chromosomal Ptrc-YFP) and UL018 (pCA2.4 Ptrc-YFP) with and without kanamycin addition. **b** Growth rate of UL006 (chromosomal Ptrc-YFP) and UL018 (pCA2.4 Ptrc-YFP) with and without kanamycin addition over the same 14-day period

**Fig. 4:**
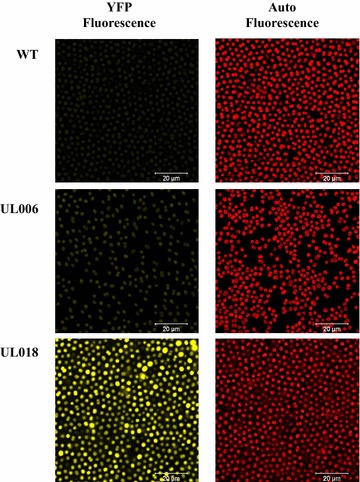
Confocal microscopy analysis of *Synechocystis* PCC 6803 WT, UL006 and UL018 cells. On the *left hand side*, representative images of cells enhanced for imaging of YFP, on the *right hand side*, the same representative images of cells enhanced for imaging of auto-fluorescence of *Synechocystis* PCC 6803

As can be seen (Fig. 2c), integration of the Ptrc-YFP cassette within pCA2.4 and the chromosomal site *psbA2* was stable; no reversion to wild-type was observed over 80 days, during which 12 sub-cultures with no antibiotic selection were carried out. No inhibitory effects on growth were observed (Fig. 3b) with both UL006 (chromosomal Ptrc-YFP) and UL018 (pCA2.4 Ptrc-YFP) growing similarly to wild-type. In addition, it was found that modification of the plasmid did not significantly affect the pCA2.4 per chromosome copy number. Significantly, YFP fluorescence from pCA2.4 was far higher than the chromosomal insertion, ranging from approximately 20 fold (day 6, without kanamycin) to 100 fold (day 14, with kanamycin added) higher YFP fluorescence detected from pCA2.4 over 14 days (Fig. 3a). For representative images of the difference in expression between chromosomal and plasmid integration site, see Fig. 4. It is likely that the difference in YFP fluorescence over the 14-day period was due to the changing copy number of pCA2.4 relative to the chromosome number during the cell cycle [16]. Overall, this analysis demonstrated that it is possible to achieve higher levels of gene expression from integration of a cassette into pCA2.4 relative to the chromosome. In addition, this analysis was carried out several times, both with and without selective pressure and yet no loss of YFP fluorescence was detected in the time period analysed indicating that integration was highly stable.

**Fig. 5:**
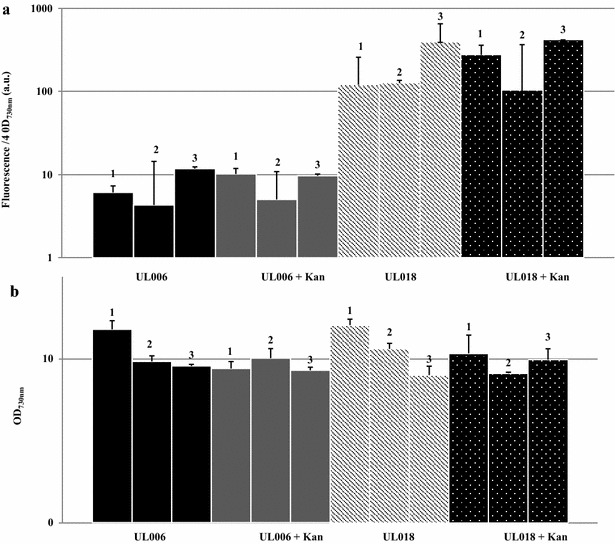
Growth rate and YFP fluorescence levels from 3 sub-cultures of UL006 and UL018. **a** Relative fluorescence measurements of three sub-cultures of UL006 and UL018 taken after 6 days growth without selective pressure (no kanamycin). **b** Optical density measurements at OD_730nm_ of three sub-cultures of UL006 and UL018 taken at the same time point

To examine the stability of integration, the growth and YFP fluorescence levels of several sequential sub-cultures of UL006 and UL018 without selective pressure were measured (Fig. 5). As can be seen, similar growth and YFP fluorescence levels were observed for each sub-culture. Therefore, it can be concluded that integration within the native plasmid may be at least as stable as integration within a chromosomal locus even though the expression level from pCA2.4 far exceeds that of a chromosomal locus.

Interestingly, in the absence of selective pressure, the overall detected YFP fluorescence level from both UL006 and UL018 was marginally reduced (Fig. 3a) yet no reversion to wild-type was detected. As all samples were normalised to the same optical density before measurement, this indicated that it is possible that the inclusion of a selective pressure may increase both the chromosomal and plasmid copy number leading to overall higher YFP fluorescence levels. Without a selective pressure, YFP fluorescence from pCA2.4 was still higher than the chromosomal insertion, ranging from approximately 17 fold (day 4) to 67 fold (day 9) (Fig. 3a). In some cases, it may in fact be more favourable to culture modified pCA2.4 strains without a selective pressure to reduce the expression level, especially if the gene being expressed causes cellular stress at very high expression levels.

## Conclusions

pCA2.4 is a stably maintained native plasmid within *Synechocystis* PCC6803. It is amenable to genetic modification and can be utilised to express selected genes at levels in excess of those achievable when compared with a chromosomal insertion. With the utilisation of an appropriate promoter, insertion into pCA2.4 may have several benefits; reduction of the time required for multi-copy modification; potentially minimisation of the requirement for antibiotics as only one modification is required to obtain multiple copies; potentially increasing stability compared to multiple chromosomal insertions as the native plasmid appears to be stably maintained. Finally, utilisation of the pCA2.4 neutral site may improve the maximum expression levels achieved with inducible promoter systems in *Synechocystis* PCC 6803 and maintain the advantages of an inducible system (Additional file 1: Figure S1).

## Methods

### Bacterial strains, DNA elements and media

The bacterial strains, plasmids and DNA elements utilised as part of this study are listed in Table 1. *Synechocystis* PCC6803 (glucose tolerant, obtained from K. Hellingwerf, UvA, Amsterdam) cells were routinely maintained at 30 °C on BG-11 media supplemented with 10 mM TES-NaOH (pH 8.2), 20 mM glucose and 0.3 % (w/v) sodium thiosulfate. All routine plasmid construction and cloning was performed in *E. coli* using Luria–Bertani (LB) broth. All media were supplemented with appropriate antimicrobial agents as required: ampicillin, 100 μg ml^−1^; kanamycin, 5–100 μg ml^−1^, streptomycin, 100 μg ml^−1^. All strains were stored at −80 °C in either Luria–Bertani (LB) broth containing 50 % glycerol (*E. coli*) or 50 % BG-11 media containing 5 % (v/v) methanol (*Synechocystis* PCC6803).

### Plasmid construction

All plasmids used in this study are listed in Table 1. All constructs were generated by utilising the In-Fusion method (Clontech, Mountain View, CA, USA) and verified by sequencing. Briefly, the In-Fusion enzyme premix was utilised to fuse PCR-generated sequences and linearised plasmid via homologous recombination at flanking 15 bp overlap sequences. The 15 bp overlap on both the PCR-generated sequences and linearised plasmids were generated via the utilisation of customised primers. The pUC18 plasmid was utilised as the backbone for all generated constructs. The YFP and β-galactosidase source plasmids are listed in Table 1. The pTRC-E* promoter sequence was obtained [18] and synthesised by Eurofins mwg/operon (85560 Ebersberg, Germany). pCA2.4 and *psbA2* homologous recombination sites were obtained by PCR with appropriate primers from AA314 [24]. The Ptrc constructs were generated by inverse PCR of a Ptrc-E* construct to delete the E* riboswitch. The P*psba2* promoter was obtained by PCR with appropriate primers from AA314. The kanamycin resistance gene was obtained from an integrative conjugative element termed ICE R391 [26].

### Transformation to Synechocystis PCC6803

*Synechocystis* PCC6803 cells were routinely cultured at 30 °C, 150 rpm, 2 % CO_2_ (Multitron Pro, Infors HT) in BG-11 liquid medium supplemented with 1 g L^−1^ HEPES–NaOH (pH 8.9) under high intensity white-light illumination (~80–100 µE m^−2^ s^−1^). Growth was monitored at OD_730nm_. Cells were grown to mid-exponential phase (OD_730nm_ of 0.4–1) and natural transformation performed as previously described with some modifications [23]. Briefly, 20 ml of cells were pelleted, washed in BG-11 media, pelleted and re-suspended in 1 ml BG-11 media to an approximate concentration of OD_730nm_ equals 10. 100 µl of cells was then mixed with 20 µl of plasmid DNA (concentration range of 100–300 ng µl^−1^) in a 1.5-ml tube. Transformations were left at 30 °C for 16 h under medium intensity white-light illumination (~20–40 µE m^−2^ s^−1^). Transformants were then plated directly onto BG-11 media containing kanamycin, 5 μg ml^−1^, ±20 mM glucose and grown for 5–10 days under medium light at 30 °C. Approximately, 40 of the resultant colonies were then streaked onto fresh BG-11 media containing increasing concentrations of antibiotic as a selective pressure to promote construct integration and full segregation. For pCA2.4 transformations, further sub-cultures to liquid BG-11 media were required to achieve full segregation. Screening for full segregation was performed by PCR. Fully segregated colonies were verified by PCR and subsequent DNA sequencing of the PCR product. The plasmid pCA2.4 modified strain (UL018) was also verified to have no chromosomal integration at the *psbA2* locus via PCR and subsequent DNA sequencing.

### Measurement of YFP fluorescence in Synechocystis PCC6803 transconjugants

All cultures were inoculated to OD_730nm_ = 0.4 and cultured with high light intensity and 2 % CO_2_ for 48 h. The culture OD_730nm_ was then measured and the equivalent of 1 ml samples normalised to OD_730nm_ = 4 were pelleted and treated with 300 µL of cell lysis buffer (Bugbuster, Cat # 70584-3, Merck Millipore) and left rocking gently for 30 min at room temperature. After lysis, samples were centrifuged at 13,000 rpm for 30 min to remove cell debris. Subsequently, samples of the supernatant were examined for fluorescence (513 nm excitation/532 nm emission) with a fluorescence spectrophotometer (Carey Eclipse, Agilent Technologies). Sample fluorescence was normalised against appropriate wild-type control samples. All reported fluorescence measurements are relative to wild-type. All data presented are the average of triplicate samples; all experiments were repeated a minimum of three separate times. For examination of strain stability without antibiotics, seed cultures were centrifuged at 4000 rpm for 15 min, washed in BG-11 media twice and re-suspended in BG-11 media ± kanamycin 50 μg ml^−1^. In addition, the average per chromosome copy number of pCA2.4 was confirmed to be similar to wild-type in UL006 and UL018 ± kanamycin according to the methods of Lee et al. 2006 [27].

### Confocal laser scanning microscopy of Synechocystis PCC6803

Cell samples were visualised using a Meta 710 laser scanning confocal microscope (Carl Zeiss, Germany) with a 63× oil immersion objective. The argon laser line (514 nm) and the helium–neon laser line (633 nm) were used for the excitation of enhanced YFP and auto-fluorescence respectively. The active spectral detection windows were configured to be 518–621 and 637–757 nm for enhanced YFP and auto-fluorescence respectively. Image processing was performed using the ZEN 2008 software package (Carl Zeiss, Germany). For each experiment, imaging parameters (laser excitation, photo multiplier tube gain, pinhole, electronic zoom size, etc.) were maintained at the same level to allow correct comparison. All cultures were inoculated to OD_730nm_ = 0.4 and cultured with high light intensity and 2 % CO2 for 72 h. The culture OD_730nm_ was then measured and all samples were normalised to the same OD_730nm_ and visualised.

## Additional file

10.1186/s13068-015-0385-x Plasmid map for pCA2.4 showing site of Ptrc-YFP construct integration into UL018. Two putative ORFs [*repA* and ORF2] are present within the native plasmid pCA2.4. P = predicted promoter, Term = predicted transcription terminator. The pCA2.4 neutral site utilised was from position 227 bp to 465 bp [GenBank ID: L13739.1].

## Abbreviations

AU: arbitrary unit
BG-11: bluegreen medium
DNA: deoxyribonucleic acid
GAL: β-galactosidase
HEPES: *N*-(2-Hydroxyethyl)piperazine-*N*′-(2-ethanesulfonic acid)
OD: optical density
ORFs: open reading frames
PCR: polymerase chain reaction
WT: wild-type
TES: *N*-[Tris(hydroxymethyl)methyl]-2-aminoethanesulfonic acid
YFP: yellow fluorescent protein
