# Supplementary figures and images for: Utilising the native plasmid, pCA2.4, from the cyanobacterium Synechocystis sp. strain PCC6803 as a cloning site for enhanced product production
Source: Biotechnol Biofuels. 2015 Dec 1;8:201. doi: 10.1186/s13068-015-0385-x (PMC4665902; doi:10.1186/s13068-015-0385-x)

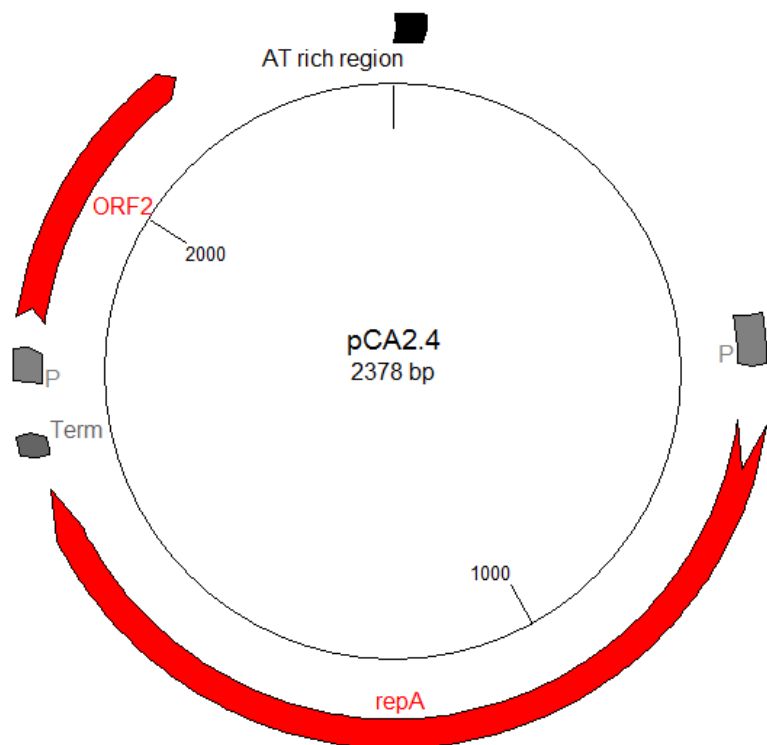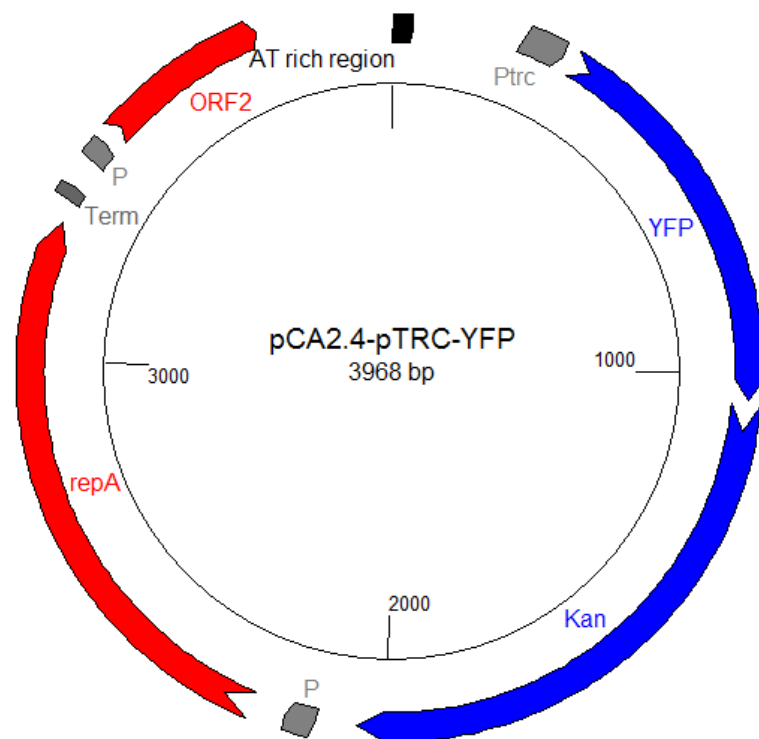

Supplement: Supplementary file 1 — 10.1186/s13068-015-0385-x Plasmid map for pCA2.4 showing site of Ptrc-YFP construct integration into UL018. Two putative ORFs [repA and ORF2] are present within the native plasmid pCA2.4. P = predicted promoter, Term = predicted transcription terminator. The pCA2.4 neutral site utilised was from position 227 bp to 465 bp [GenBank ID: L13739.1]. [file 13068_2015_385_MOESM1_ESM.pdf]
